# Gelatin- and Papaya-Based Biodegradable and Edible Packaging Films to Counter Plastic Waste Generation

**DOI:** 10.3390/ma15031046

**Published:** 2022-01-29

**Authors:** Jaweria Ashfaq, Iftikhar Ahmed Channa, Asif Ahmed Shaikh, Ali Dad Chandio, Aqeel Ahmed Shah, Bushra Bughio, Ashfaque Birmahani, Sultan Alshehri, Mohammed M. Ghoneim

**Affiliations:** 1 Department of Metallurgical, Materials & Environmental Engineering, NED University of Engineering and Technology, Karachi 75270, Pakistan; Jaweriaashfaq72@gmail.com (J.A.); asif.shaikh@neduet.edu.pk (A.A.S.); aqeelshah@neduet.edu.pk (A.A.S.); 2 Larkana Campus, Shaheed Mohtarma Benazir Bhutto Medical University, Larkana 77150, Pakistan; Bushra.bughio1@gmail.com; 3 Ojha Campus, DOW University of Health Sciences (DUHS), Karachi City 74200, Pakistan; dentistashfaque007@gmail.com; 4 Department of Pharmaceutics, College of Pharmacy, King Saud University, Riyadh 1145, Saudi Arabia; Salshehri1@ksu.edu.sa; 5 Department of Pharmacy Practice, College of Pharmacy, AlMaarefa University, Ad Diriyah 13713, Saudi Arabia; mghoneim@mcst.edu.sa

**Keywords:** edible film, papaya puree, gelatin, soy protein, film properties, thin film, food packaging

## Abstract

Most of the food packaging materials used in the market are petroleum-based plastics; such materials are neither biodegradable nor environmentally friendly and require years to decompose. To overcome these problems, biodegradable and edible materials are encouraged to be used because such materials degrade quickly due to the actions of bacteria, fungi, and other environmental effects. In this work, commonly available household materials such as gelatin, soy protein, corn starch, and papaya were used to prepare cost-effective lab-scale biodegradable and edible packaging film as an effective alternative to commercial plastics to reduce waste generation. Prepared films were characterized in terms of Fourier transform infrared spectroscopy (FTIR), water vapor transmission rate (WVTR), optical transparency, and tensile strength. FTIR confirmed the addition of papaya and soy protein to the gelatin backbone. WVTR of the gelatin-papaya films was recorded to be less than 50 g/m^2^/day. This water vapor barrier was five times better than films of pristine gelatin. The gelatin, papaya, and soy protein films exhibited transparencies of around 70% in the visible region. The tensile strength of the film was 2.44 MPa, which improved by a factor of 1.5 for the films containing papaya and soy protein. The barrier qualities of the gelatin and gelatin-papaya films maintained the properties even after going through 2000 bending cycles. From the results, it is inferred that the prepared films are ideally suitable for food encapsulation and their production on a larger scale can considerably cut down the plastic wastage.

## 1. Introduction

The quality of food is the most important factor for its manufacturing and selling points of view [1]. Food starts to degrade when it encounters the environment or has any interaction with ambient air. This is because the ambient air comprises humidity as well as oxygen and both are detrimental to organic stuff [2,3,4,5,6]. Packaging plays an important role in protecting and extending the shelf life of food [7]. The prime features of packaging materials are low oxygen and moisture permittivity in ambient conditions, inertness towards food material, and chemical and mechanical stability in prescribed environmental conditions as per ASTM E460 [8,9]. Most of the materials used in the food packaging industry are non-biodegradable [10]. A non-biodegradable material is a material that is not easily decomposed but can cause pollution and clogging [11]. The most utilized non-biodegradable plastics are polyethylene (PE), polypropylene (PP), polyethylene terephthalate (PET), polytrimethylene terephthalate (PTT), and polyamide (PA) [12]. All these types of plastics, on one hand, are easy to manufacture, but, on the other hand, pose serious environmental safety-related issues such as long-term degradation rate and damage to natural ecosystems [13]. For these reasons, the use of environmentally friendly materials as alternatives to non-biodegradable plastic packaging is unavoidable. In recent years, edible as well as biodegradable non-plastic packaging films have been used as substitutes for standard plastics [14]. Biodegradable materials are usually made of biopolymers, which are regularly found in living organisms, such as cellulose and protein. Such materials disintegrate readily due to the operations of bacteria, fungus, and other living creatures [15]. Biodegradable substances are often found in everyday life, such as food waste, tree leaves, and grass clippings. Various forms of packaging, such as foils, bags, boxes, etc., are made from biodegradable polymers, which means these materials are safe to pack food items [16].

Guerrini et al. [17] reported that the conventional plastics used in agriculture and food packaging purposes have a very short lifespan; hence these plastics need to be used within six months of their manufacturing. As a result, a huge amount of plastic waste is generated that needs extra care for its proper disposal. It is estimated that 30% of plastic waste generated comes from agriculture and packaging with a high risk of lagging agricultural system contamination [9,18]. Hence, the use of biodegradable materials is required, as they are suitable because of their physicochemical and mechanical properties to replace traditional plastics, thereby reducing the waste generation. As per the international standards, biodegradable materials can be left directly in the soil, where they are degraded by microorganisms and environmental actions [19,20].

As Jeevahan et al. [21] proposed, edible materials can also be used for packaging purposes, and such materials can straightforwardly be ingested by people or animals with no health hazards and thereby can significantly cut down waste generation. Edible polymers are classified as polysaccharides, proteins, and lipids [22,23]. There are several types of polysaccharides, such as cellulose, gum, starch, and chitosan, but starch has become the most common and particularly important polysaccharide material in edible and biodegradable films due to its low cost, flexibility, and clarity [24,25,26,27]. Proteins can include animal extracted proteins (casein, gelatin, milk, egg white, etc.) and plant extracted proteins (wheat gluten, man, soy, protein, and rice). Two materials are commonly used as protein materials in edible films: gelatin and soy protein. Edible animal extracted gelatin has abundant sources and is easy to form films compared to plant extracted gelatin. Edible animal gelatin is high in protein and has a characteristic amino acid composition that confers several health advantages as well as film formability [28,29]. Soy protein food film has shown biodegradability and bio-composite properties. In terms of barrier properties, soy protein film has a higher water vapor transmission rate (WVP) value than most synthetic polymers, and it has inherent hydrophilicity [23,24]. Common lipid compounds used to make edible films include glycerin, neutral lipids, fatty acids, waxes, and resins [29]. Papaya puree and all fruits contain natural wax. Papaya puree is rich in pectin, which can be used as the basis for making a biodegradable and environmentally friendly edible film [25,30]. Glycerol is a hydrophilic plasticizer. When added correctly relative to the biopolymer content, it can reduce the intermolecular forces and increase the fluidity of the polymer chain, a process commonly used to improve the mechanical properties of edible films. These types of biopolymers help to make edible and degradable films [31,32].

In addition to this, Tulamandi et al. worked on the polysaccharides-based edible films extracted from papaya (PP). These investigations were based on the physical and mechanical properties of films created from the mixtures of papaya with gelatin (G). In their study, film-forming solutions of various levels of papaya puree, gelatin, and skim soy protein were prepared, and the films were casted at room temperature. The films exhibited a tensile strength of around 8.20 ± 0.02 MPa and tear strength of 0.73 ± 0.001 g/μm. On the other hand, when the defatted soy protein was added together with gelatin to papaya puree, the film showed a significant increase in water contact angle of 78.14°. These films also exhibited oxygen and moisture permeability of around 100 g/m^2^/day [25].

Based on work by Tulamandi et al., the purpose of this work is to create a path for the production of edible packaging films on a larger scale by using natural ingredients and simple coating methods. The main idea is to make edible films generalized in a way to replace conventional plastics so that waste generation can be significantly reduced. Furthermore, mechanical characteristics are elaborated and discussed in this work to compare their practical applications in real life. The developed films are also analysed in terms of moisture permeability and transparency to meet packaging requirements. As the packaging materials should also be resistant to UV light while maintaining transparency in the visible region, the use of papaya is promoted because it has natural UV blocking capability. Additionally, this work also focuses on the economical film-forming methods to keep the processing cost low.

## 2. Materials and Methods

### 2.1. Materials

The following materials were used for the elaboration of the edible films: Papaya puree, prepared from the papaya fruit, which was purchased from the local fruit market in Karachi (Pakistan) at maturity stage 3 (26–50% of yellow skin). The glycerin (SKU-dis_26618), having a purity level of 99.97 %, was supplied by Biosynth Pharma (Pvt) Ltd., Karachi, Pakistan. The corn starch (UPC 620514019192) was supplied by National Foods Co., Ltd., Karachi, Pakistan. Nutrena (Karachi, Pakistan) supplied defatted soy protein (SKU-182212900), which had less than 1% oil and was employed as a source of protein in the edible film creation. Gelatin (Cert no. 70401) was purchased from Rossmoor Food Products Pvt Ltd., Karachi, Pakistan.

### 2.2. Preparation of Film

**Figure 1:**
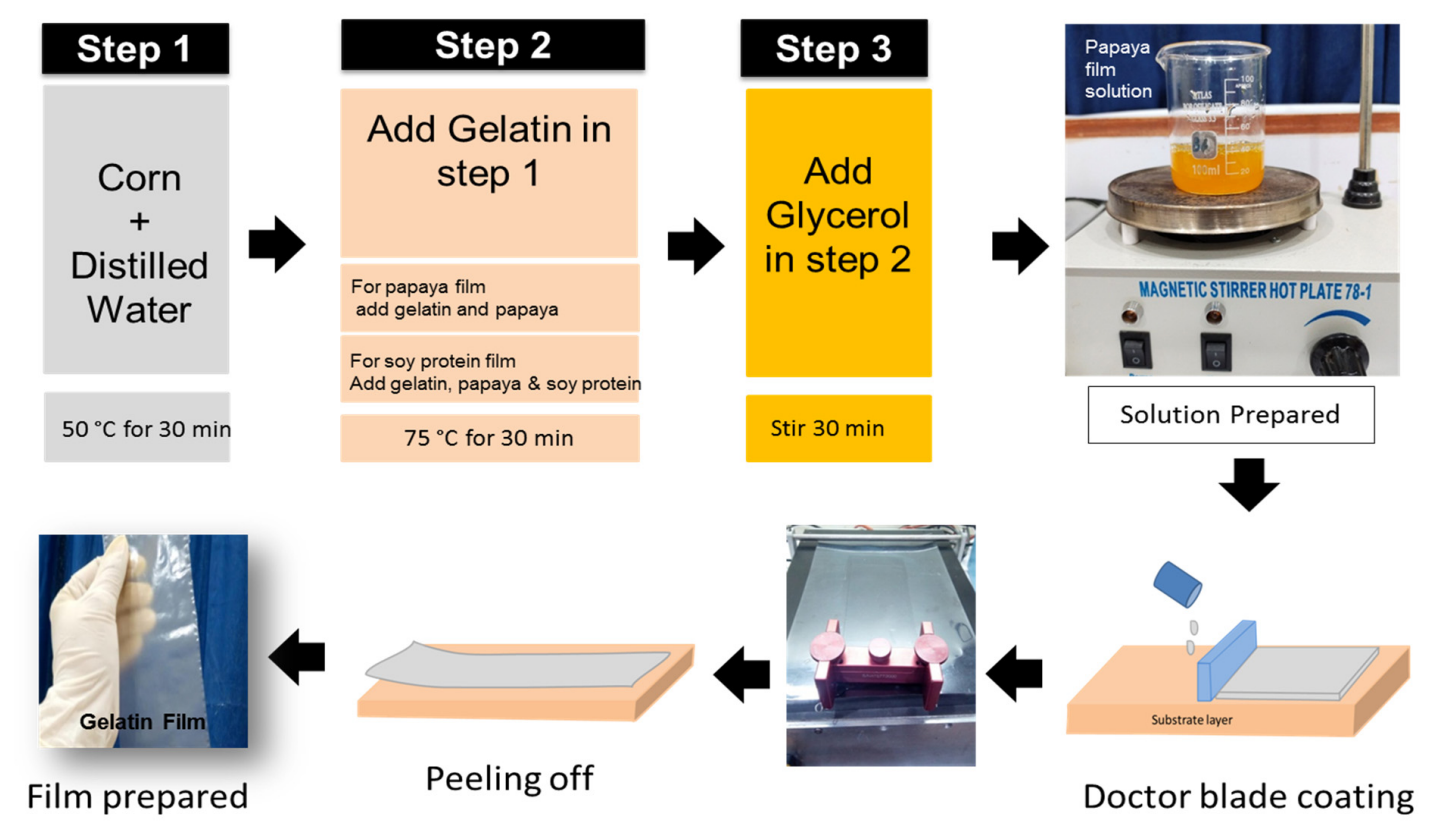
Schematic diagrams of the process flow for the preparation of solution and processing of edible films.

The film-forming solution was prepared in three steps as shown in Figure 1. In the initial step, 2 wt.% of corn starch was dissolved in 50 mL of distilled water for 30 min at 50 °C. In the 2nd step, gelatin was added to the solution and stirred for 30 min at 75 °C. In the final step, 3 wt.% of glycerin was added as a plasticizer to each kind of film solution and was constantly stirred for 30 min via a magnetic stirrer to generate solutions. To investigate the effect of papaya on gelatin films, 8:10 *w*/*w* g papaya was added to the G-2 solution before plasticization to generate G/PP-1 and G/PP-2 papaya/gelatin blended film solutions. To investigate the effect of defatted soy protein on papaya/gelatin films, 2:4 *w*/*w* defatted soy protein was added to G/PP-2 to produce G/PP/SP-1 and G/PP/SP-2. A total of six formulations was obtained, as shown in Table 1.

Edible films were formed utilizing a doctor blade (ZAA 2300, Zehntner Testing Instruments, Switzerland) for forming films on 210 mm × 297 mm transparent PET substrates (Melinex^®^ ST504, DuPont Teijin Films UK Ltd., Middlesbrough, UK). The solution was dried for 24 h at 40 °C. The films were peeled off and free-standing films were obtained (see Figure 1). These films were stored in an inert atmosphere until the samples were further characterized.

### 2.3. Film Characterization

#### 2.3.1. FT-IR Analysis

The edible films were analyzed by an FT-IR device (Bruker ALPHA-P, Karlsruhe, Germany) with a wavelength range of 500 cm^−1^ to 4000 cm^−1^. Spectra were obtained using 64 scan summations at 4 cm^−1^ resolutions.

#### 2.3.2. UV-VIS

Transparency of the samples was analyzed by a UV-VIS spectrophotometer (Shimadzu UV-1800, Shimadzu Deutschland GmbH, Duisburg, Germany) at a wavelength between 200 and 800 nm.

#### 2.3.3. Contact Angle

The contact angle was measured by contact angle goniometer SL200A manufactured by KINO Scientific Instrument Inc., Boston, MA, USA by using water droplets.

#### 2.3.4. Tensile Test

Each film’s tensile strength (TS) and elongation at break (EAB) in machine and cross directions were measured by using a Z005 Zwick/Roell universal testing machine from Germany. The samples were prepared under the ASTM D 882-10 standards, with an initial grasp separation of 50 mm and a crosshead speed of 5 mm/min. The films were cut into strips, 30 mm wide by 130 mm long. The mechanical characteristics of the samples were determined using a 5N load cell. Each film’s stated results were the average of at least three measurements.

#### 2.3.5. Bending and Hardness

The edible film was bent using an in-house-designed cyclic curve analyzer with one end stationary and the opposing end moving straight to and fro, cycling the boundary film in a modified twisting range. The hardness of edible film was determined by using nano-hardness (Anton Paar nanoindentation hardness tester having a diamond indenter), with a maximum load of 10.0 mN applied at a rate of 20 mN/min. For the whole film, the poison ratio was 0.30. The nanoindentations were performed as per ISO 14577, and at least five indentations were performed for each film.

#### 2.3.6. WVTR

Thwing-Albert Instrument Company (West Berlin, NJ, USA) provided a standard aluminum cup with a diameter of 6.35 cm that complies with ASTM standard E-96. The test was performed as per the method mentioned in Channa et al. [33].

## 3. Results and Discussion

### 3.1. FT-IR

**Figure 2:**
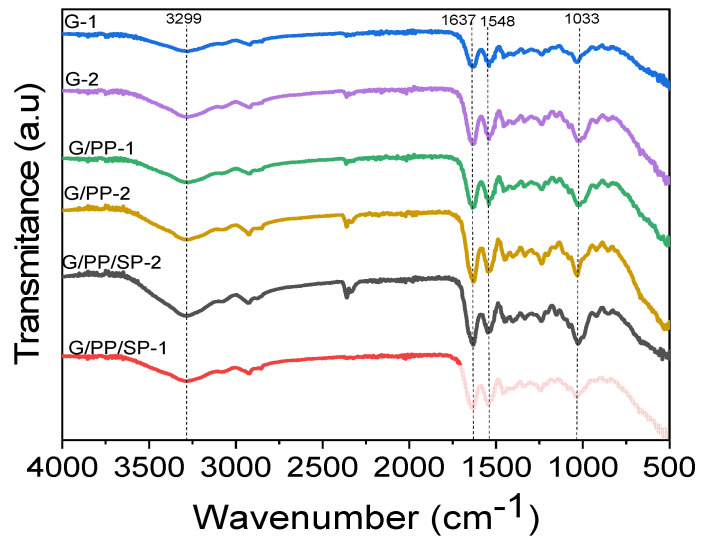
FT-IR transmission spectra of edible films and their composites with gelatin concentration of 6:9 *w*/*w* (indicated by blue and violet colors), papaya concentration of 8:10 *w*/*w* (indicated by green and brownish-yellow colors), and soy protein concentration 2:4 *w*/*w* (indicated by black and red colors) in the range of 4000–500 cm^−1^.

FT-IR investigation was performed to obtain a profound view of the chemical structure of the prepared films [34]. The FT-IR results are shown in Figure 2. The bonding of papaya puree, gelatin, and defatted soy protein composite films is demonstrated by these transmittance peaks [25]. FT-IR spectra of edible films showed that the addition of gelatin and soy protein to papaya did not change the molecular interactions of the resulting films, but it decreased the intensity of the peak slightly. All spectra showed three primary regions, and the results are under the work conducted by K. Wang et al. [35]: (i) A wide asymmetric band in the range of 3500 and 2800 cm^−1^: Peaks around 2931 cm^−1^ encompassing the characteristic –C–H vibration peaks are associated with the methylene hydrogen molecules. A wide peak in the range of 3000–3500 cm^−1^ is the result of stretching vibrations of the –OH groups [36]. (ii) An area somewhere in the range of 1700 and 1100 cm^−1^ is normally for the amide groups: All films displayed trademark ingestion peaks at around 1637, 1457, and 1240 cm^−1^, which relates to –C=O stretching. (iii) An absorption region somewhere in the range of 800 and 1200 cm^−1^: The groups at 1020–1036 cm^−1^ bands are based on –C–O extending vibrations. Peak intensities are quite comparable with maize starch-gelatin composite films [37]. In addition, for the G/PP/SP-2 film, the highest intensity of peaks is present in the region of the –OH group at the level of 3298 cm^−1^. This is because the interaction between papaya, soy protein, and gelatin can form hydrogen bonds in the film. G/PP/SP-2 increased bond energy, resulting in increased maximum strength [38].

### 3.2. Light Transmission Rate and Transparency

Food quality is highly dependent on protection from UV rays. Oxidation changes the taste of food, reduces its nutritional value, and creates toxic compounds, all of which can make food sources less satisfactory or unsuitable to shoppers. Oxygen produced by the ultraviolet irradiation of the sun is a rapid cause of lipid oxidation. Even at very low temperatures, the oxidation rate is greatly increased and the quality of food processed and stored is deteriorated [39]. The UV screening capacity of food packaging film is desirable to prevent the oxidation of UV light. Biopolymer films have been utilized to stay away from food quality deterioration due to physical compound changes or synthetic responses. As of late, gelatin has been utilized because of its great barrier properties [40]. Different parts can likewise be added, for example, soy protein into the biopolymer network to upgrade the usefulness, quality, stability, and safety of packaging food sources [41].

**Figure 3:**
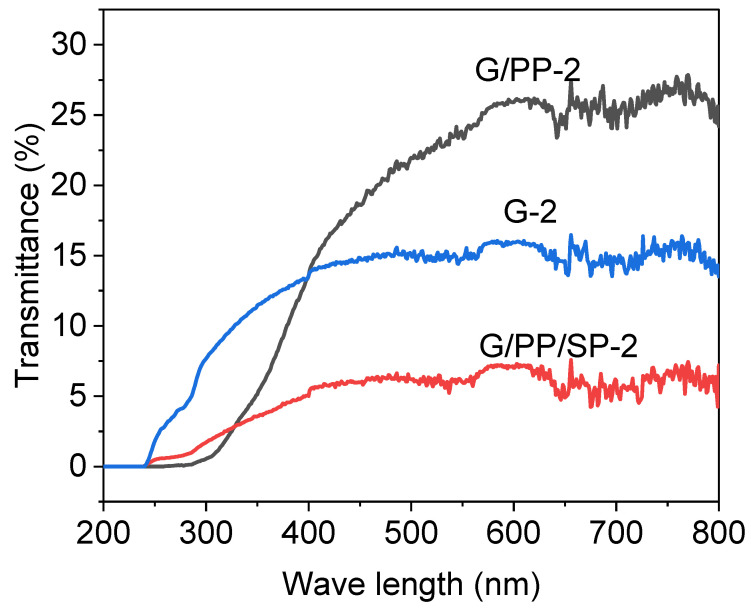
Transmittance spectra of edible films (black line indicates G/PP-2 film, blue line indicates G-2 film, and red line indicates G/PP/SP-2 film).

The properties of the film to block ultraviolet (UV) and visible light (Vis) were measured, and the blocking properties of the film were measured at different wavelengths (200 to 800 nm) using dual-beam UV-VIS spectroscopy. They are represented by low transmittances between 200 and 350 nm and 400 and 800 nm, respectively. The results of UV-VIS spectroscopic analysis are shown in Figure 3. UV transmittance is appreciated as it increases the shelf life of packaged foods. High transparency of the container in the visible area is required since it allows the consumer to visually verify the condition of the product. The figures show the transmission spectrum in the spectral range from 280 to 600 nm. Papaya film had a higher transmittance in the visible region (660 nm) than in the UV region (280 nm) because papaya has properties of being a natural UV barrier. The addition of soy protein to the film significantly affected and reduced the UV transmission of the film (280 nm). The G/PP/SP-2 film lowered the transmittance of the film to UV light (280 nm). The combination of glycerol and gelatin reduced the transmittance. The transmittance of the G/PP-2 film sample is the highest, which means that the papaya does not have the same high UV protection properties as it does for the VIS region [34].

**Figure 4:**
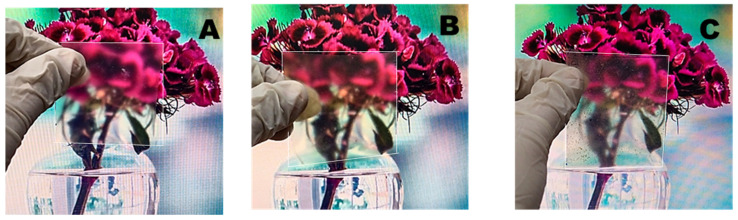
Transparency of edible film (white line indicates the film area): (**A**) gelatin film, (**B**) papaya gelatin film, (**C**) soy protein papaya gelatin film.

Film transparency is vital in foodstuffs, as it affects light-sensitive products. The transparency of edible films is a significant tactile part of edible films and coatings that must be accepted by consumers [42]. The transparency of the film was confirmed at a wavelength of 500 cm^−1^. Film G/PP-2 had higher transparency compared to other films, and film G/PP/SP-2 had lower transparency (Table 2 and Figure 4). The higher rate of the G/PP/SP2 film delamination was primarily responsible for the lower transparency. Higher DSP concentrations increase particle size and compactness while decreasing film transparency [43]. Apple starch films incorporated with ellagic acid have quite similar transparencies [44]. The structure of the polymer and the bonding that develops during film formation determines its transparency. In addition, the film on the airside is more glossy, darker, and rougher than the film on the carrier side [45].

### 3.3. Contact Angle

**Figure 5:**
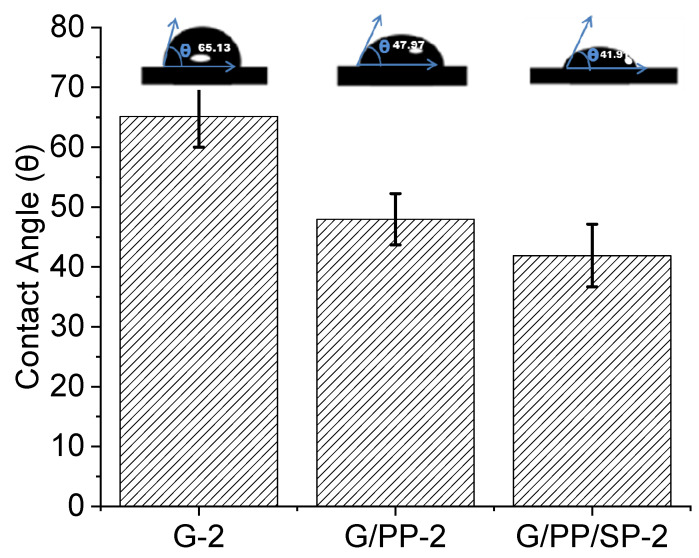
Hydrophilic nature of the edible film, showing water droplet shape as well as measured corresponding contact angles (CA) of G-2, G/PP-2, and G/PP/SP films.

Table 3 shows measurements of contact angles for various pure liquids in film samples. Low contact angle demonstrates that the strong surface presents hydrophilic attributes, while high contact points uncover hydrophobic properties. When PP and DSP were added, the WCA value decreased, as shown in Table 2 (and in Figure 5) [46]. The WCA of papaya films was comparable with soy protein isolate, polyvinyl alcohol, and glycerol blend films [47,48]. Water contact point (θ) θ < 95 represents hydrophilic properties and θ > 95 indicates hydrophobic properties [49]. Contact angle values were observed to be slightly higher on the airside than on the film support side, as was also observed in studies of papaya and defatted soy protein films. The lowest values were observed for the DSP base film, but with the addition of PP in gelatin base film, the WCA significantly increased from 39° to about 47°, respectively (Table 2). No distinctions were seen between the contact points of tests with various thicknesses [45].

### 3.4. Tensile Test

**Figure 6:**
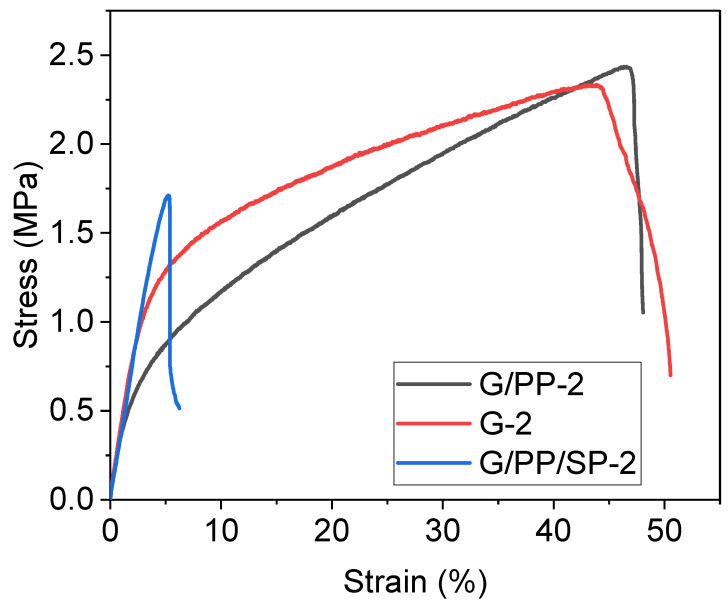
Stress–strain diagram of edible film with different concentration of gelatin (red line), papaya (black line), and soy protein (blue line).

The most researched mechanical properties of edible film are tensile strength and elongation break [50]. The upsides of these mechanical characteristics are introduced in Table 4. Figure 4 demonstrates strain behavior for the edible gelatin-consistent film with variable contents of papaya and soy protein under the impact of tensile tension. In the beginning, there was a quick increase in stress for all the edible films tested, and the elastic deformation was linearly proportional to the strain. After this step, the food material was reached and the plastic deformation began. The stress behavior–strain charts in the plastic phase of the edible film specimens vary. The stress–strain graphs in Figure 6 demonstrate that the tensile strength of the edible films decreases while ductility increases when the soy protein levels increase [51]. Elongation at break (%E) of investigated films went from 48.9 to 39.8 MPa and 6.2 to 50.5% individually and relied upon the kind of polymer utilized which is similar to LPDE [52]. Without defatted soy protein, the addition of papaya to the gelatin films essentially expanded TS and arrived at a limit of 48.9 MPa. Gelatin has a direct design and a restricted monomer component, which leads to good film-forming properties. When defatted soy protein was added to papaya films, within the high concentration of gelatin, the TS diminished while EAB additionally declined extraordinarily. Perhaps with the addition of papaya puree in gelatin films (without defatted soy protein), the TS of film expanded fundamentally, which shows that papaya adds to the increment in TS of the gelatin films. The increase in tensile strength was regulated, not only for the geometry and the hardness of film but also due to the formation of a rigid, continuous network of hydrogen bonding with papaya fruit [53]. When both the defatted soy protein and papaya were added to the gelatin films, a desperate pattern was noticed, i.e., TS decreased significantly. Unexpectedly, the gelatin films added to defatted soy protein and papaya puree showed diminished EAB, likely because of the decrease in protein sub-atomic versatility by cross-connecting [25].

Elongation break measures the flexibility of a film composed of G, DSP, and PP. When the concentration of defatted soy protein was increased in the gelatin film, the elongation rate was reduced. The quantity of papaya in the gelatin film increased the elongation break value. G2 film also exhibited a strong elongation break. Gelatin had the greatest influence on film stretch, and EB increased with the G content in composite films [43].

### 3.5. Bendability

**Figure 7:**
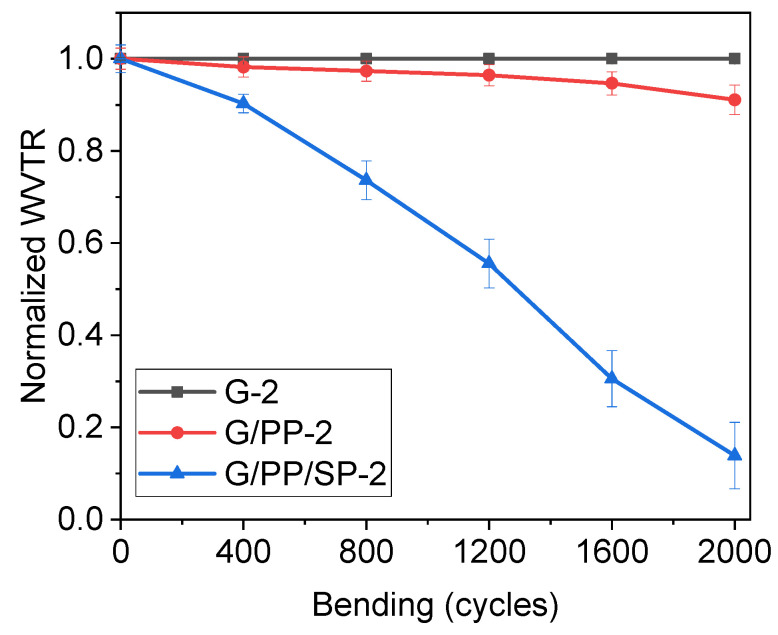
Normalized WVTR of edible films vs. the number of bending cycles with a bending radius of 6.35 cm. The black curve represents gelatin (G-2) film, the red curve represents G/PP-s film, and the blue curve represents G/PP/SP-2 films, and the thickness of each tested film is about 100 µm.

One of the most important criteria for edible films is flexibility. Because of the protein–polysaccharide interaction and the plasticizing impact of glycerin with papaya, gelatin, and soy protein, materials become more flexible. This phenomenon might also be attributed to the possibility of increased molecular affinity. To assess the loss of barrier quality, the edible films were exposed to bending cycles at a radius of 6.5 cm. After the bending test, there was no visible damage to the G-2 and G/PP-2 films. In order to assess the bending effect on films, all films were subjected to WVTR testing. Similar methodology was adapted as mentioned in our previous work [33]. Figure 7 shows the normalized WVTR values of the films after various bending cycles. After being bent, the edible films G-2 and G/PP-2 always returned to their original positions and did not lose internal chain adhesion. In less than 600 bending cycles, the G/PP/SP-2 films started to shatter and lose adhesion in the film matrix, which resulted in WVTR degradation and exhibited 80% degradation as compared to their initial WVTR. G-2 and G/PP-2 almost retained their initial WVTR after 2000 bending cycles.

### 3.6. Hardness

**Figure 8:**
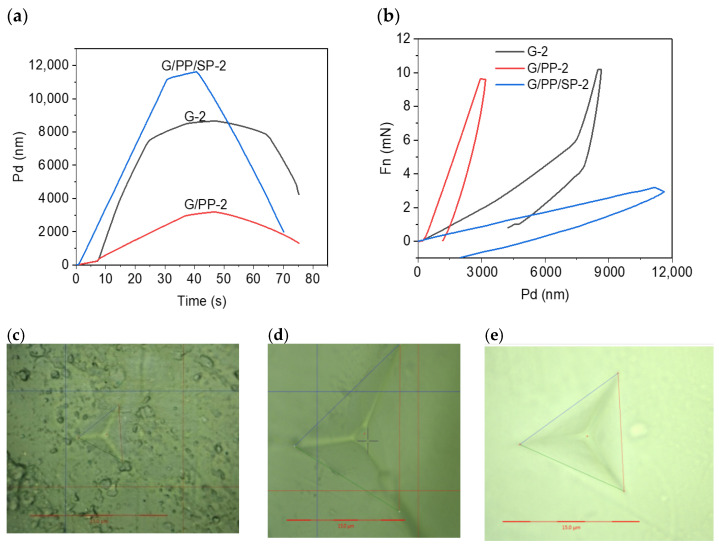
Nanoindentation graphs of the films: (**a**) Penetration depth vs. time curves of the edible thin films, with gelatin (G-2) film indicated by blue line, papaya (G/PP-2) film indicated by red line, and soy protein (G/PP/SP-2) film indicated by black line. (**b**) Nanoindentation curve of edible films plotted against force vs. displacement. Microscopic images of nanoindentation impressions of (**c**) G-2 films, (**d**) G/PP-2 films, and (**e**) G/PP/SP-2 films.

Nanoindentation is a useful technique that can be used for a comprehensive mechanical analysis of thin films and coatings. Nanoindentation is also considered an alternative to macroscale mechanical characterizations [51]. Figure 8a shows the time vs. the penetration depth of the indenter, whereas Figure 8b shows the force vs. the penetration depth. It is revealed from the indentation curves that the lowest surface hardness was found in G/PP/SP-2, as the penetration was higher in the samples (~12,000 nm) when around 3 mN of force was applied (Figure 8a,b). The corresponding indentation image is shown in Figure 8e, whereas sample G-2 (simple gelatin film) exhibited the lowest penetration depth, i.e., 3000 nm, even when a force of 10 mN was applied (Figure 8a,b), and the corresponding indentation is shown in Figure 8c. The films of G/PP-2 showed the penetration depth which lies between the penetration depth of the G-2 and G/PP/SP-2. This result suggests that the films of G/PP-2 are neither as soft as G/PP.SP-2 nor hard like G-2 (the corresponding indentation is shown in Figure 8d). This result is also in line with the result obtained via bulk tensile strength analysis, as mentioned previously in Table 4. The possible cause of this result could be higher content of papaya as well as starch in the gelatin film. This result is also in accordance with the study carried out by Tulamandi et al. [25]. In their study, they concluded that the linear papaya chains prefer to interact with hydrogen bonds, resulting in stronger films. Additionally, they also concluded that the starch chains maintain molecular mobility and hence are the main reason for maintaining the hardness. Another possible reason for lower hardness of G-2 and G/PP/SP-2 films could also be surface roughness. The surface roughness values of these films were found to be 0.14 µm and 0.49 µm, respectively. Furthermore, according to Kadhim et al. [51], surface roughness and surface nano-hardness are negatively linked with each other; a smooth surface may yield a harder surface [51,54].

### 3.7. Water Vapor Transmission Rate

**Figure 9:**
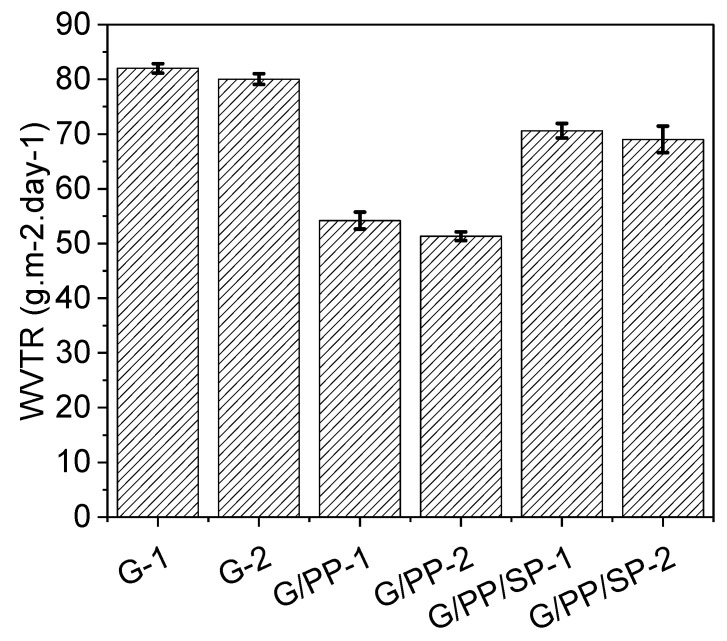
Effect of gelatin, papaya, and soy protein on water vapor transmission rate (WVTR) of edible films.

Water plays an important role in food spoilage; therefore, an important property of edible films is their ability to prevent the exchange of moisture between the medium and the food matrix. WVTR is the measure of the moisture that goes through a unit space of material per unit time [42]. When the papaya content of the composite film increased from 8 to 10%, the WVTR decreased significantly because of its easy dispersion in the porous film, as presented in Table 4 and Figure 9. The value of WVTR for all edible films increased when compared with the taro starch in addition to glycerol, a result from Siskawardani et al. [55]. As a result, the denser membrane structure created more tortuous pathways for the diffusion of water molecules and thus reduced the membrane’s WVTR. On other hand, when soy protein was added to the papaya and gelatin films, the increased carboxyl group yielded increased WVTR values. The film surface became coarser and thicker due to the high concentration of soy protein, with increased pores in the membrane matrix resulting in bigger openings in the film grids [56]. The results also showed that the WVTR of the edible gelatinous film was slightly higher than that of papaya, which may be due to the water-induced swelling of the membrane [57].

Plasticizer likewise plays a significant part in WVTR. The concentration of glycerol plasticizer can increase the flexibility and water vapor transmission rate (WVTR) of edible films. The use of plasticizers increases the physical and functional properties of edible films, (1) flexibility, sensitivity, and moisture, and changes (2) functional properties. Plasticizer decrease influences: (1) the biopolymer chains adjacent to each other, (2) the sensitivity that occurs out of the water, and (3) the flexibility of the material [58].

At room temperature, minimal weight loss was observed with 10 g papaya edible film (3.6 gm), and maximum weight loss was observed with 9 gm gelatin (5.76 gm). In general, the results show that weight loss can be reduced by adding various content to slow the penetration of water into the environment, where the content itself acts as a barrier to the edible surface [59].

## 4. Conclusions

The development of biodegradable and edible materials to replace conventional plastics is gaining attention as public awareness of health and environmental problems is growing. In this study, we created gelatin-based, biodegradable, and edible synthetic polymer films using simplified and cost-effective film-forming methods. The prepared films were tested by various means such as chemical analysis, transparency moisture permeability, mechanical strength, hardness, and wettability. FT-IR analysis was performed for its chemical compositional study. FT-IR data revealed that addition of papaya and soy protein to gelatin solution did not change the original chemistry of the gelatin films, as no significant change was observed in FT-IR spectra after addition of papaya and soy protein to gelatin films. However, the transparency of gelatin films was enhanced by around 10% by addition of papaya, and the same was reduced by 20% when soy protein was mixed in gelatin together with papaya. This could be due to surface imperfections generated which may scatter the light, causing overall transparency reduction. Tensile strength analysis revealed that the G/PP-2 film exhibited the highest Young’s modulus of elasticity, which is comparable to commercial plastic films, indicating gelatin-based films can easily replace conventional plastic packaging films. Moisture permeability values were found to be around 50 g/m^2^.day in ambient conditions, which is in accordance with food grading packaging standards. Furthermore, the films of gelatin and gelatin-papaya showed excellent bendability, as they retained the WVTR after 2000 bending cycles, whereas films of G/PP/SP-2 showed fast degradation (around 80% as compared to their initial performance) after 2000 bending cycles. This could be due to their brittle nature. The lab scale production cost (calculated from raw material usage) of gelatin-papaya films is less than 0.5 USD/m^2^. Therefore, overall, gelatin and gelatin mixed with papaya exhibited the best performance in most tests and can be perfect choices for large-scale production.

## Figures and Tables

**Table 1:** Composition of samples along with the sample identification acronyms.

Sample	Composition
G-1	Gelatin (6 wt.%) + Corn Starch (2 wt.%) + Glycerin (3 wt.%)
G-2	Gelatin (9 wt.%) + Corn Starch (2 wt.%) + Glycerin (3 wt.%)
G/PP-1	Gelatin (9 wt.%) + Papaya (8 wt.%) + Corn Starch (2 wt.%) + Glycerin (3 wt.%)
G/PP-2	Gelatin (9 wt.%) + Papaya (10 wt.%) + Corn Starch (2 wt.%) + Glycerin (3 wt.%)
G/PP/SP-1	Gelatin (9 wt.%) + Papaya (10 wt.%) + Soy Protein (2 wt.%) + Corn Starch (2 wt.%) + Glycerin (3 wt.%)
G/PP/SP-2	Gelatin (9 wt.%) + Papaya (10 wt.%) + Soy Protein (4 wt.%) + Corn Starch (2 wt.%) + glycerin (3 wt.%)

**Table 2:** Optical and wetting properties of edible films.

Sample	T (%)	CA (°)
G-2	15.20	65.13 ± 5.13
G/PP-2	25.9	47.97 ± 4.28
G/PP/SP-2	6.08	41.91 ± 5.23

**Table 3:** Mechanical properties of edible films.

Sample	Young’s Modulus (MPa)	Tensile Strength (MPa)	Elongation at Break (%)	Indentation Hardness (MPa)	Vickers Hardness (MPa)	Bending Cycle
G-2	45.9	2.33	48.1	6.5875	610.07	2000
G/PP-2	48.9	2.44	50.5	67.287	6231.5	2000
G/PP/SP-2	39.8	1.71	6.2	1.7431	161.43	<560

**Table 4:** Barrier properties of edible film.

Sample	Thickness (µm)	WVTR (g·m^−2^·Day^−1^)	Weight Loss (gm)
G-1	102.85 ± 4.87	82.01 ± 0.856	5.75
G-2	97.142 ± 3.06	80.05 ± 1.003	5.65
PP-1	118.57 ± 6.26	54.19 ± 1.553	3.8
PP-2	107.14 ± 4.87	51.34 ± 0.788	3.6
SP-1	131.42 ± 2.08	70.60 ± 1.321	4.95
SP-2	175.71 ± 5.34	69.03 ± 2.411	4.84

## Data Availability

Data available upon request from the corresponding authors.
